# Spontaneous Resolution of Post-COVID-19-Associated Sacral Neuromodulation Device Dysfunction and Overactive Bladder Symptoms: A Report of Two Cases

**DOI:** 10.7759/cureus.71722

**Published:** 2024-10-17

**Authors:** Ryan Wong, Morgan R Sturgis, William A Langbo, Sarah A Adelstein

**Affiliations:** 1 Department of Medicine, Dr. Kiran C. Patel College of Osteopathic Medicine, Nova Southeastern University, Fort Lauderdale, USA; 2 Department of Urology, Rush University Medical Center, Chicago, USA

**Keywords:** covid-19, implant, overactive bladder, sacral neuromodulation, urinary incontinece

## Abstract

Sacral neuromodulation (SNM) is an effective third-line therapy for overactive bladder (OAB). COVID-19 may have a temporary negative effect on SNM function. We describe two cases of increased sensory thresholds and loss of SNM efficacy following COVID-19 infection. In both of these cases, SNM efficacy, sensory thresholds, and impedances were spontaneously restored after a few months without surgical intervention. Withholding surgical revision due to transient post-COVID-19-induced SNM impairment may be appropriate; however, further research is needed to elucidate the mechanism involved with COVID-19 interaction with SNM functionality.

## Introduction

There is a substantial number of patients who recovered from COVID-19 that have persistent sequelae referred to as post-COVID-19 conditions (PCC). Several studies have indicated that COVID-19 may play a role in genitourinary symptoms associated with an overactive bladder (OAB), as highlighted in a recently termed condition known as COVID-19-associated cystitis, which is similar to OAB [1].

Sacral neuromodulation (SNM) is an effective third-line therapy for OAB. After implantation, loss of efficacy can occur because of changes in the symptoms/functionality or because of a change in SNM functionality (e.g., end of neurostimulator battery life or neuroelectrode damage). Device interrogation with evaluation of impedances across all combinations of neuroelectrode circuits is one way to evaluate neuroelectrode integrity and functioning. Known causes of abnormal impedance include open circuitry, where there is an incomplete connection to the neurostimulator due to lead fracture, or short circuitry from traumatic injury to the neuroelectrode.

Herein, we report two patients from routine follow-ups in one academic urology practice who were noted to have a decline in their SNM function within months of COVID-19 infection with spontaneous resolution. We aim to describe their clinical courses and also report on related literature.

## Case presentation

In routine follow-ups in one academic urology practice with a high volume of SNM implants, two out of 34 patients implanted with SNM in the past five years described loss of efficacy after COVID-19 infection. They described having worsened frequency/urgency symptoms and were noted to have an increase in sensory thresholds. These findings spontaneously resolved after several months without surgical revision.

The first patient is a 75-year-old female with OAB and genitourinary syndrome of menopause. She had her InterStim device (Medtronic, Minneapolis, MN) implanted in July 2019. While she was noted to have shooting sensations with her program (P) 1, her symptoms resolved after 10 months. Three years post-op, she continued to have normal impedances with five to six daily voids (Table 1). In April 2023, the patient presented to the emergency room with urinary tract infection symptoms and was subsequently diagnosed with COVID-19. One-month post-COVID-19 infection, she reported increased urinary frequency and abnormal impedances. The patient also reported an increase in daily voids (16-20) from baseline. On InterStim interrogation, an abnormal impedance value of >4,000 ohms were found on e3. Normal impedance values were found on e0-2. All programs using e3 were inactivated and three custom advanced programs (ea-c) were created using e0-2 only (Table 1). With three months of observation and conservative management, the patient’s impedance values spontaneously returned to normal, and her urinary frequency returned to baseline.

**Table 1:** Patient symptoms, management, and sensory thresholds (ST)

		Pre-COVID-19	Post-COVID-19	Post-COVID-19
Patient 1 (75 yo F)	SNM Sensory Thresholds (*left on)	8/2022	5/2023 (1 month post-COVID)	8/2023 (4 months post-COVID)
		P1 0.3 mA vaginal*	P3 0.8 mA vaginal*	Pa 0.8 mA vaginal
		P2 0.4 mA vaginal	Pa 1.1 mA vaginal	P1 1.1 mA vaginal*
		P3 0.2 mA vaginal	Pb 1.1 mA vaginal	
		P4 0.5 mA vaginal	Pc 1.0 mA vaginal	
		Normal impedances	Abnormal impedance on e3 (>4000 ohms)	Normal impedances
	Daily voids	5-6	16-20	6-7
	Incontinence (pads/day)	0	0	0
Patient 2 (58 yo F)	SNM Sensory Thresholds (*left on)	11/2020	6/2021 (5 months post-COVID-19)	9/2021 (8 months post-COVID-19)
		P1 1.7 mA vaginal*	P1 2.8 mA vaginal*	P1 1.2 mA vaginal
		P2 1.6 mA rectal	P2 2.5 mA rectal	P2 1.2 mA vaginal
		P3 1.7 mA perineal	P3 2.5 mA rectal	P3 0.4 mA vaginal*
		P4 1.5 mA rectal	P4 2.2 mA rectal	P4 0.9 mA rectal
		P5 1.9 mA rectal		
		Normal impedances	Normal impedances	Normal impedances
	Daily voids	5-6	14-16	7-8
	Incontinence (pads/day)	4	6	3

The second patient is a 58-year-old female with urgency predominant mixed urinary incontinence. She had her InterStim device implanted in October 2020. Impedance values were tested and found to be normal one-month post-op in all four leads. She had a baseline void of five to six times per day and was noted to have persistent urinary incontinence (Table 1). In December 2020, the patient was diagnosed with COVID-19. Five months after resolution of COVID-19 infection, the patient had an increased sensory threshold in all four leads while impedance values remained normal. These were associated with severe worsening of urinary frequency (14-16 voids per day), nocturia, and increased incontinence. After three months of observation and conservative management, the patient’s InterStim sensory thresholds across all leads and symptoms spontaneously returned to baseline.

## Discussion

We highlight two patients that described SNM device loss of efficacy after COVID-19 infection, that later spontaneously resolved without surgical intervention. These two cases presented out of 34 patients implanted with SNM in the past five years at one academic urology practice with a high volume of SNM implants.

SNM has been shown to be highly efficacious for management of OAB. ARTISAN-SNM trial, a recent prospective multi-center study, found 93% of patients (n=121) to be therapy responders, including 37% who reported no incontinence, at two-year follow-up [2]. For a typical patient who has undergone SNM implantation for OAB, standard sensory thresholds are between one and two volts [3]. As seen in the second patient we present, initial sensory thresholds were set in this range. For our first patient, however, she had sub-sensory thresholds as her baseline. Sub-sensory thresholds have been reported to be as efficacious in reducing urgency incontinence when compared to standard sensory thresholds [4]. This indicates that changes in a patient's baseline sensory threshold suggest SNM loss of efficacy and in our patients, PCC elicited this.

A comprehensive literature review was performed to identify similar cases of COVID-19-associated impairment of neuromodulation devices. There are currently no documented reports of PCC on SNM function. However, one report highlighted two cases of COVID-19-induced cochlear implant failure [5]. The etiology was found to be device short circuit as a result of COVID-19. Another case report described a case of COVID-19 induced pacemaker capture failure [6]. The mechanisms underlying PCC and nervous system dysregulation interplay in these cases may be similarly implicated in the two patients we describe herein, although this is unclear without further research.

While the literature failed to reveal any prior case that discussed COVID-19 infection effects on SNM device functionality, a retrospective cohort study found that patients who had symptomatic COVID-19 infection were more likely to have worsening OAB symptoms [7]. About 20% of those cases reported were de novo OAB. Another study corroborated these results by highlighting an increase in symptom score, defined by the American Urological Association (AUA) OAB assessment tool, and increased quality of life score, whereby a higher score is indicative of dissatisfaction, following recovery from acute stage COVID-19 infection [8]. Recently, a study investigated the impacts of long COVID-19 on COVID-19-associated cystitis [1]. Conservative management of new onset COVID-19-associated cystitis was found to decrease OAB scores and increase the quality-of-life scores. Thus, conservative management for patients with SNM failure due to COVID-19 may also be indicated.

Ultimately, it is also unknown whether our patients’ SNM failure may reoccur. Research into PCC is still at its nascent stages and its long-term effects are important to guide clinical management of OAB. Nevertheless, investigation is needed to elucidate the effects of the PCC axis on SNM.

## Conclusions

COVID-19 may have a temporary adverse effect on SNM function. In both of these patients’ cases, SNM efficacy, sensory thresholds, and impedances were restored after a few months without any surgical intervention. While the mechanism of impedance resolution is not perfectly clear, withholding operative revision due to transient PCC SNM impairment may be appropriate. However, it is important to consider the trade-offs between waiting for the resolution of incontinence symptoms and the impact on the patients' quality of life versus undergoing surgery.
